# Supplementary material for: Perception of Pathologists in Poland of Artificial Intelligence and Machine Learning in Medical Diagnosis—A Cross-Sectional Study
Source: J Pers Med. 2023 Jun 7;13(6):962. doi: 10.3390/jpm13060962 (PMC10301272; doi:10.3390/jpm13060962)
Supplement: Supplementary file 1 [file jpm-13-00962-s001.zip › jpm-2379656-supplementary.pdf]

Supplementary data - Questionnaire

**Demographic data**		
Q1	Age	
Q2	Years of medical practice experience	
Q3	Gender	
Q4	Specialty	Allergy and Immunology<br>Diagnostic Radiology<br>Emergency medicine<br>Internal medicine<br>Nephrology<br>Medical genetics<br>Oncology<br>Pathology<br>Preventive medicine<br>Pediatrics<br>Surgery<br>Urology<br>Other
**Artificial intelligence and Machine learning**		
Q5	Did you use any artificial intelligence or machine learning models before?	Yes<br>No
Q6	How much do you trust the artificial intelligence/Machine Learning results in a scale-out of 10?	0 = I do not trust<br>5 = Neutral<br>10 = Fully trust
Q7	How do you evaluate the artificial intelligence/Machine Learning in diagnosing cancer cells on a scale-out of 10	0 = It cannot diagnose it<br>5 = Neutral<br>10 = Full diagnosis
Q8	I have knowledge about artificial intelligence /machine learning in medical diagnosis.	Strongly agree<br>Agree<br>Neither agree nor disagree<br>Disagree<br>Strongly disagree
Q9	Artificial intelligence /Machine learning has valuable applications in the medical field.	Strongly agree<br>Agree<br>Neither agree nor disagree<br>Disagree<br>Strongly disagree
Q10	The diagnostic ability of artificial intelligence /Machine learning is better than the clinical experience of a human doctor.	Strongly agree<br>Agree<br>Neither agree nor disagree<br>Disagree<br>Strongly disagree
Q11	Artificial intelligence /machine learning approaches will save time and money for physicians.	Strongly agree<br>Agree<br>Neither agree nor disagree<br>Disagree<br>Strongly disagree
Q12	Artificial intelligence could replace my work in future	Strongly agree<br>Agree<br>Neither agree nor disagree<br>Disagree<br>Strongly disagree
Q13	Artificial Intelligence /Machine learning can speed up processes in medical diagnosis.	Strongly agree<br>Agree<br>Neither agree nor disagree<br>Disagree<br>Strongly disagree
Q14	Artificial Intelligence /Machine learning can help reduce medical errors	Strongly agree<br>Agree<br>Neither agree nor disagree<br>Disagree<br>Strongly disagree
Q15	Artificial Intelligence /Machine learning can deliver many high-quality data in real-time.	Strongly agree<br>Agree<br>Neither agree nor disagree<br>Disagree<br>Strongly disagree
Q16	Artificial Intelligence /Machine learning has no space-time limitation	Strongly agree<br>Agree<br>Neither agree nor disagree<br>Disagree<br>Strongly disagree
Q17	Artificial intelligence could have enough information/algorithms to provide opinions on difficult cases.	Strongly agree<br>Agree<br>Neither agree nor disagree<br>Disagree

Supplementary data - Questionnaire

		Strongly disagree
Q18	Artificial intelligence is applicable to every patient	Strongly agree<br>Agree<br>Neither agree nor disagree<br>Disagree<br>Strongly disagree
Q19	Artificial intelligence is difficult to apply to controversial subjects	Strongly agree<br>Agree<br>Neither agree nor disagree<br>Disagree<br>Strongly disagree
Q20	Artificial intelligence has a low ability to sympathize emotional well-being of the patient.	Strongly agree<br>Agree<br>Neither agree nor disagree<br>Disagree<br>Strongly disagree
Q21	If your medical diagnosis differs from artificial intelligence's diagnosis, which will you follow?	Doctor's opinion "your opinion/your supervisor"<br>Artificial intelligence's opinion<br>Patient's choice
Q22	In which field of medicine do you think artificial intelligence will be most helpful	Making a diagnosis<br>Making treatment decisions<br>Direct treatment (including surgery)<br>Biopharmaceutical research and development<br>Providing medical assistance in underserved areas<br>Development of social insurance program<br>Other
Q23	Who do you think will be liable for legal problems caused by artificial intelligence?	Doctor in charge<br>The company that created the artificial intelligence<br>The patient who consented to follow artificial intelligence's input<br>Other
Q24	In 25 words minimum, for co-words qualitative purposes, kindly share your general thoughts about AI and ML in medical diagnosis, including but not limited to your visions, concerns, personal experience..etc	
